# Simian Immunodeficiency Virus-Infected Macrophage Traffic Out of the Central Nervous System

**DOI:** 10.21203/rs.3.rs-6435804/v1

**Published:** 2025-05-20

**Authors:** Xavier Alvarez, Zoey K. Wallis, Cecily C. Midkiff, Jaclyn Mallard, Pete J. Didier, Kenneth C. Williams

## Abstract

We use intracisternal (i.c.) injection of fluorescent-superparamagnetic iron oxide nanoparticles (SPION) in SIV-infected monkeys that labeled CNS CD68-CD163-CD206 perivascular, meningeal, and choroid plexus (CP) macrophages. SPION + CD163 + macrophages are also found in the optic nerves, nasal septum, and cribriform plate - potential sites-paths of macrophage exit. Outside the CNS CD163 + SPION + macrophages labeled in the CNS are in deep cervical lymph node (dCLN), spleen, dorsal root ganglia (DRG). CD163 + SPION + CNS macrophages are abundant 24 hours p.i. in normal animals and decrease over time without CNS inflammation, but accumulate with SIV infection and inflammation. Greater numbers of SPION + macrophages traffic out of the CNS in normal animals and decrease with infection. Productively infected, SPION + macrophages are found in the CNS and dCLN, spleen, and DRG of infected animals 7–14 days post i.c. injection. These data are consistent with SPION + macrophages, some of which are viral infected, trafficking out of the CNS.

### Summary

SPION+ macrophages can leave the CNS normally but accumulate with SIV.  SPION+ SIV+ are found in the CNS, at CNS borders, including optical nerve, and cribriform plate, and are outside the CNS in dcLN and dorsal route ganglia.

### Introduction

The central nervous system and CNS are HIV infected early and late post-infection in the periphery even with ART(Williams et al., 2001, Zink et al., 2010). HIV and SIV are detected early in the CNS and is associated with perivascular cuffs of macrophages; and later in CNS lesions with macrophage accumulation(Williams et al., 2001, Bednar et al., 2015, Lackner et al., 1991). We and others have shown that HIV and SIV are found within CNS perivascular macrophages, meningeal macrophages, and choroid plexus macrophages(Nowlin et al., 2015, Williams et al., 2001). The biology of these cells is important to consider with regard to the CNS as a viral reservoir. The origins of monocytes and macrophages includes bone marrow cells that traffic to the CNS normally and those derived from embryonic stem cells (yolk sac-derived) that repopulate within the CNS by cell division(Bechmann et al., 2001a, Goldmann et al., 2016, Lassmann et al., 1993, Hickey et al., 1992). Moreover, the biology of CNS inflammation, macrophage traffic in and out of the CNS normally and with HIV infection, is of critical interest.

The majority of HIV and SIV-infected cells in the CNS are blood-derived, perivascular macrophages that are located at the blood-CSF interface. These macrophages are a key component of the CNS immune defense and, when infected, can become reservoirs for SIV and HIV(Williams et al., 2001, Kierdorf et al., 2019). Once infected CNS macrophages can facilitate viral persistence and dissemination in the CNS and contribute to neuroinflammation and the development of neurocognitive disorders(Saylor et al., 2016, Williams and Hickey, 2002). The cerebrospinal fluid (CSF) is another route of CNS viral dissemination where infected cells travel through the bloodstream and migrate into the CNS either as part of normal repopulation or in response to infection(Bednar et al., 2015, Clements et al., 2005, Zink et al., 1999). The trafficking of SIV or HIV-infected macrophages into the CNS is well-documented, but whether these cells can traffic out has significant implications for understanding HIV and SIV persistence and latency, as well as the challenges in eradicating these infections and is less well understood.

Counter to the dogma that the CNS lacked or had limited draining lymphatics(Proulx, 2021), it is now well established that CNS fluids—and in some instances cells—can drain or exit into the periphery(Aspelund et al., 2015, Engelhardt et al., 2016, Hatterer et al., 2006, Iliff et al., 2012, Kida et al., 1993, Ma et al., 2017, Proulx, 2021). Radiolabeled proteins and fluorescence dyes injected intracisternally and fluorescent-labeled antigen-presenting cells (APCs) transplanted into the CNS have been detected in deep cervical lymph nodes (dcLN)(Hatterer et al., 2008) likely having been trafficked via meningeal lymphatics. Such studies were done in normal non-inflamed or infected animals and in brain injury or autoimmune models(Da Mesquita et al., 2018, Louveau et al., 2018, Louveau et al., 2017). Importantly, studies in rodents and non-human primates have demonstrated CNS myelin antigen-presenting macrophages and dendritic cells (APC) in lymph nodes with CNS myelin proteins, suggesting such cell traffic is important for maintaining tolerance to CNS antigens and autoimmune responses (experimental allergic encephalomyelitis (EAE) and multiple sclerosis)(Laman and Weller, 2013, van Zwam et al., 2009, de Vos et al., 2002). Indeed, ablation of the meningeal lymphatics reduced clinical symptoms in EAE(Louveau et al., 2018). An alternative route for cells to exit the CNS is via the central canal and meninges of the spinal cord that connect the inner layers of the peripheral nerve root sheaths as they leave the spinal cord(Ma et al., 2019, Ligocki et al., 2024). This forms a direct connection near the root attachment, the subarachnoid angle, resulting in a continuous passage linking the cerebrospinal fluid-filled subarachnoid space (SAS) of the brain and spinal cord with the perineural space of the nerve roots(Ma et al., 2019, Jacob et al., 2019, Ligocki et al., 2024). This path communicates with the dorsal root ganglia (DRG) at the interface of the CNS, the peripheral nervous system (PNS), and immune organs outside the CNS(Jacob et al., 2019, Miura et al., 1998, Hu and McLachlan, 2002). Prior studies, using transplanted and/or injected fluorescent-labeled cells, demonstrate they can leave the CNS. Such experimental rodent models are important and informative, but they have the caveat of inducing CNS injury, breach of the blood-brain barrier (BBB), or both, that can facilitate CNS inflammation (Hatterer et al., 2006, Hatterer et al., 2008, Aspelund et al., 2015). Importantly, such studies have not been done to date in humans or non-human primates or with HIV or SIV infection.

In this study, we injected superparamagnetic iron oxide nanoparticles (SPION) in normal and SIV-infected monkeys and studied their uptake and distribution within the CNS over time, and SPION + CNS macrophage traffic out of the CNS. Due to their size (0.86μm average diameter), SPION do not passively diffuse out of the CNS but are instead taken up by CNS macrophages in perivascular spaces, the meninges, and the choroid plexus from where they can exit. SPION + macrophages peak in the CNS 24 post ic inoculation and decrease with time in normal animals but accumulate in the CNS with SIV infection. SIPON + macrophages are found outside the CNS in dCLN and DRG, and there are greater numbers of these cells with normal animals than SIV infected animals. SPION + and SIV-RNA and gp41

+ SPION + macrophages are found outside the CNS in infected animals. Studies in this paper are the first to utilize i.c. SPION injections demonstrate the labeling, accumulation, and trafficking out of macrophage populations in infected animals. These findings underscore the ability of SPION + macrophages, labeled in the CNS, to traffic to peripheral lymph organs and the peripheral nervous system. These data are discussed in the context of CNS inflammation and resolution of infection, and HIV viral reservoirs.

## Results

We injected SPIONs (33 mg/ml) intracisternally (i.c.) into normal non-infected and SIV-infected animals and studied their uptake within CNS macrophages one hour (dextran and SPION), 1 day (dextran and SPION) and 7-, 14-, and 28-days post injection (Fig. 1). SPION-labeled cells were detected by bright-field microscopy, Prussian blue iron stain, or using the internal fluorescence of the SPION (data not shown) (Fig. 2). Multiple SPION were taken up per labeled cells that are distributed within perivascular spaces, the meninges, and the choroid plexus (Fig. 2). We do not find free (non-cell-associated) SPION in the CNS likely due to their size (0.86µm diameter) but scattered free SPION are found around meninges surrounding the brain and the DRG in the subarachnoid space contiguous with the central canal of the spinal cord and CSF (data below). Immunohistochemical (Prussian blue) and fluorescence (Dragon green) SPION are consistently taken up by CD68, CD163, and CD206 perivascular macrophages, meningeal macrophages, and choroid plexus (CP) macrophages (Fig. 3). There is a consistent > 2-fold increase in the number of SPION + meningeal versus SPION + perivascular macrophages, consistent with the greater overall number of total meningeal versus perivascular macrophages in each respective compartment (Table 1). It is likely also that different fluid dynamics of CSF in the meninges versus than in the perivascular space(Srinivas et al., 2021, Brown et al., 2020, Ma et al., 2017) account for differential SPION uptake in meningeal versus perivascular macrophages. Over time, there are decreased numbers of SPION + perivascular ($p = 0.06$) and meningeal macrophages ($p = 0.09$) in non-infected animals consistent with SPION-labeled cells leaving the CNS (Table 2). In contrast, in SIV-infected animals, there are increased numbers of SPION + macrophages in perivascular space and meninges consistent with CNS inflammation and macrophage accumulation (Table 2).

**Table 1. Accumulation of SPION+ macrophage in the CNS over time.** Data are cell numbers (average+/-StDev) counted in whole tissue sections and expressed as cells per $mm^2$. The total area examined is indicated and expressed as tissue area per $mm^2$. The area of the meninges and parenchyma were obtained by tiling and hand-drawing around each ROI. The total number of SPION+ macrophages counted is shown in parentheses. A minimum of 4 sections per animal were examined. Prism was used for graphing and statistical analysis Kruskal-Wallis nonparametric test.

| Time with SPION | Non-Infected | SIV+ |
|---|---|---|
| **Perivascular Macrophages** | | |
| 7 Days | 0.3 ± 0.3<br>A= 652 mm²<br>(155) | 1.6 ± 0.7<br>A= 608 mm²<br>(936) |
| 14 Days | 0.6 ± 0.8<br>A= 675 mm²<br>(122) | 1.8 ± 1.9<br>A= 792 mm²<br>(916) |
| 28 Days | 0.2 ± 0.1<br>A=252 mm²<br>(51) | 0.6 ± 0.5<br>A= 558 mm²<br>(302) |
| **Meningeal Macrophages** | | |
| 7 Days | 81 ± 43<br>A= 18 mm²<br>(1331) | 63 ± 63<br>A= 15 mm²<br>(1362) |
| 14 Days | 36 ± 33<br>A= 12 mm²<br>(420) | 25 ± 14<br>A= 32 mm²<br>(538) |
| 28 Days | 50 ± 47<br>A= 7 mm²<br>(85) | 125 ± 111<br>A= 6 mm²<br>(868) |

**Table 2. SPION+ macrophage outside the CNS over time.** Data are cell numbers (average+/-StDev) counted in n=10 fields of view at 40x magnification expressed as the number of cells per mm$^2$.

| Tissue Outside of the CNS | Days after SPIONs Injection in the Cisterna Magna | | | | |
|---|---|---|---|---|---|
| | 0.4 | 1 | 7 | 14 | 28 |
| **Non-Infected Animals** | | | | | |
| Optic Nerve+Eye | nd | nd | 20 ± 6.6 | 2.7 ± 2.8 | 49 ± 18 |
| Cervical Lymph Node | nd | nd | 24 ± 2.3 | 0.35 ± 0.63 | 4.8 ± 4.1 |
| Nasal Septum | nd | nd | 147 ± 12 | nd | 1.3 ± 1.4 |
| Cribiform Plate | nd | nd | 6.0 ± 2.5 | nd | 49 ± 41 |
| Spleen | nd | nd | 73 ± 48 | 66 ± 38 | 49 ± 18 |
| **SIV+ Animals** | | | | | |
| Optic Nerve+Eye | 0 | 0 | nd | 0.67 ± 0.92 | 3.3 ± 3.8 |
| Cervical Lymph Node | 0 | 0 | 0.43 ± 0.06 | 2.8 ± 2.9 | 1.1 ± 0.22 |
| Nasal Septum | 0 | 0 | 2.5 ± 0.51 | 0.33 ± 2.7 | 0 |
| Axiliar Lymph Node | 0 | 0 | 0 | 1.7 ± 2.1 | 1.1 ± 0.77 |
| Spleen | 0 | 0 | 0.26 ± 0.27 | 58 ± 43 | 12 ± 10 |

Next, we studied SPION + macrophages exiting the CNS. We find SPION + macrophages in the optic nerve, nasal septum, cribriform plate, at the interface border of the CNS (border associated macrophages, BAM), and outside the CNS in the superficial and dcLN, spleen, and dorsal root ganglia (cervical, thoracic and lumbar, DRG) 1-, 7-, 14-, and 28-days post SPION inoculation in non-infected and SIV infected animals (Fig. 4). To assess the distribution of cells and fluid leaving the CNS, we injected soluble fluorescence-labeled dextran and SPION together in a subset of animals (i.c.) (n = 2). Fluorescently labeled dextran dye is found in the dcLN 1 hour after i.c. inoculation in the medullary sinuses, but SPION + macrophages are not detected until 24 hr post i.c. inoculation and is in the paracortex (Fig. 5). The 24-hour time lag between finding injected soluble-dextran versus SPION + macrophages is consistent with macrophage uptake of SPION in the CNS and their traffic out to the LN. In addition to dcLN, SPION + positive macrophages are found within the optic nerve, cribriform plate, axillary LN, spleen, and DRG (cervical, thoracic, lumber) 7–28 days post-inoculation in non-infected animals that have no CNS inflammation (Table 2). SPION + macrophages within the optic nerves are in the perineurium and within the nerve fascicles (Wallis, et al. 2024 under review). In addition, SPION + macrophages in the choroid plexus are located in the stroma and within Kolmer macrophages on the apical side (Wallis, et al. 2024). With SIV infection there are decreased numbers of SPION + macrophages in peripheral sites and a trend of accumulation in the CNS (Table 2). Overall, these data, similar to the observations in the CNS suggest that SPION-labeled macrophages leave the CNS at a higher rate in non-infected animals but accumulate in the CNS with SIV infection and CNS inflammation.

Using the internal fluorescence of SPION and andti-SIVgp41 and SIV-RNA probes, we find SPION + SIVgp41 + and SIV-RNA + macrophages in the CNS, and outside the CNS in dcLN in SIV infected animals, and SIV infected animals with AIDS (Fig. 6). Serial section and double label IF, show these are CD163 + SIVgp41+ (Fig. 6). In AIDS animals with SIVE, we find SPION + SIV-RNA + macrophages (20 to 50 SPION + SIV-RNA + cells per dcLN section, 1.0 ± 1.7 SPION + SIV-RNA+/$mm^2$) inside of the CNS and in the dcLN (Fig. 6). These data are consistent with SPION-labeled macrophages that were in the CNS that are productively SIV-infected. Whether these are SIV-infected in the CNS and carried SIV-RNA and DNA out, or if they were infected upon leaving the CNS is not determined in this study.

## Discussion

In this study we injected SPION i.c. and assessed their distribution within the CNS over time in normal and SIV infected animals. We found SPIONS in PV macrophages, macrophages in the meninges, and CP that are CD163, CD206, CD68, and in some cases Iba1 positive. There were a greater number of SPION-labeled macrophages within the meninges than the PV macrophages, likely a consequence of greater number of total macrophages in the meninges and also the rate of fluid speed and SPION interaction with macrophages in the meninges versus the PV space (Srinivas et al., 2021, Brown et al., 2020, Ma et al., 2017). Additionally, macrophages in the meninges consistently had larger numbers of SPION than PV macrophages which may alter their macrophage to traffic (Delcroix et al., 2009, Jendelová et al., 2004). This is likely to the different biology of macrophages in the meninges versus the perivascular space where meningeal macrophages are DC myeloid-like versus innate macrophages in the perivascular space (Williams and Burdo, 2012, Bechmann et al., 2001b). We found that the ratio of total macrophages versus SPION + macrophages in each compartment are relatively fixed in non-infected and SIV-infected animals. Outside the CNS we found SPION + macrophages in dcLN, far fewer in superficial LN, and found them in the spleen and DRG with greatest numbers in the DRG, consistent with the DRG being continuous with the subarachnoid space of the spinal cord and in contact with CSF into which the SPION were injected (Ligocki et al., 2024). In non-infected animals, there were higher numbers of SPION + macrophages 7 days after injection that normalized over time. With SIV infection, the number of SPION + macrophages in the CNS increased with the development of lesions and macrophage accumulation, and fewer SPION + macrophages were found that trafficked out. Importantly, almost all of the SPION in the CNS and outside were within macrophages and not ones that simply diffused out most likely due to the size of the SPION (Carare et al., 2008). Others using SPION injected i.c. have similar findings (Hatterer et al., 2006, Hatterer et al., 2008). Our findings suggest that with SIV infection and macrophage accumulation in the CNS, there is an accumulation of SPION + macrophages. In contrast, in non-infected animals, the SPION + macrophages seem less likely to accumulate in the CNS and increased numbers are found outside of the CNS consistent with traffic out. Interestingly, in the present study, we find with ART more SPION + macrophages are found outside the CNS and fewer are inside, consistent with ART resolving CNS inflammation and more SPION + cells trafficking out (Wallis et al., submitted).

In SIV infected animals, we found SPION + macrophages in the CNS and importantly outside the CNS in dcLN. In this study, we cannot ascertain whether they were infected within the CNS or outside. Work by our group and others use gene flow analysis and SIV sequencing demonstrate viral traffic from the CNS to tissues outside late in infection (Salemi et al., 2005, Lamers et al., 2011). Such analysis would need to be done on a single cell level comparing CNS to peripheral viral sequences.

Studies in rodents using transplanted or injected (CSF) cells and dyes into the CNS support the notion that immune cells including DC, T cells and B cells and in some cases, monocytes can leave the CNS (Hatterer et al., 2006, Hatterer et al., 2008, Kida et al., 1993, Goldmann et al., 2006, Karman et al., 2004, Laaker et al., 2023). Those studies by the nature of injection and injury somewhat compromise the BBB and activated the brain (Hatterer et al., 2006, Hatterer et al., 2008). Our study reported here is the first to study macrophage traffic out to the periphery with SIV infection. Pathways by which cells can traffic out are many and have been described and reviewed by others (Laaker et al., 2023, Raper et al., 2016, Lamers et al., 2016, Da Mesquita et al., 2018, Frederick and Louveau, 2020, Kanamori and Kipnis, 2020, das Neves et al., 2021). These include meningeal lymphatics and perineural pathways via the cribriform plate (Goldmann et al., 2006, Hatterer et al., 2006, Hatterer et al., 2008). It is interesting in this study that i.c. injected dextran are readily found outside the CNS in dcLN hours after administration but SPION + macrophages are not detected until 24 hours post i.c. inoculation. This is likely because of the size restriction of the SPION that do not readily leak out (Daneman and Prat, 2015, Chen and Konofagou, 2014, Iliff et al., 2012) but are instead taken up by macrophage before they leave in contrast to dextran dyes that readily leak out. Also, the location of the SPION in the dcLN is different than dextran dye, suggesting differential pathways of fluid versus myeloid cell traffic (Laman and Weller, 2013, Hatterer et al., 2006, Girard et al., 2012).

We and others have previously demonstrated in HIV-infected humans and SIV-infected monkeys that PV macrophages are a major site in the CNS of productive lentiviral infection (Williams et al., 2001, Nowlin et al., 2015, Abreu et al., 2019). In other work (Wallis et al., submitted 2024) we find ART effectively clears the CNS perivascular macrophage SIV infection and the majority of CNS meninges and CP macrophages, and following 4 weeks of ART interruption, CNS perivascular macrophages remain SIV—RNA negative but there is a rebound in the number of SIV-RNA + meningeal macrophage and CP macrophages (Wallis, et al., 2024). Similarly, in the periphery, plasma virus is decreased, as it is in the spleen and dcLN, but interestingly not in the DRG. We and others have shown that the DRG appears to be less resistant to ART with macrophage accumulation and persistent activation despite ART (Wallis, et al., under review AJP, 2024)(Warfield et al., 2023).

In this study, we now also show the macrophages in the CP also are targets. Others have previously noted early CP macrophage infected by HIV and SIV with aseptic meningitis but have generally not considered this to be a reservoir site (Falangola et al., 1994, Hanly and Petito, 1998, Chen et al., 2000). This however seems to be the case and might be a source of CSF virus as macrophages in the CP are close to both blood and CSF production, that subsequently baths the brain and spinal cord (Falangola et al., 1994, Hanly and Petito, 1998, Chen et al., 2000, Lackner et al., 1991, Liu et al., 2000). Another site of interest is the DRG part of which is at the interface of the CNS and the PNS. SPION are abundant in the DRG that is in communication with the subarachnoid space of the spinal cord, and the subarachnoid angle surrounding the nerve sheath (McCabe and Low, 1969, Hu and McLachlan, 2002). We find SPION within macrophages in the epineurium, and with fascicles of the nerve, suggesting a novel route of possible traffic of macrophages (Wallis et al., under review AJP, 2024). Importantly macrophages with SIV in the peripheral nerve and DRG are less affected by ART, unlike the spleen and dcLN. Thus, the DRG and peripheral and cranial nerves, which are still affected with HIV in PLWH on durable ART, deserve more investigation with regard to viral reservoirs and macrophage. It is important to note that Clements et al., show evidence of replication competent virus in monocytes of PLWH on ART (Abreu et al., 2019, Veenhuis et al., 2023).

Possible short comings of our work include our use of CD8 depletion for a rapid AIDS model with high tissue pathology. Because of the CD8 depletion ART in this model, for this 3-4-month time period does not lower plasma virus to non-detectable levels. That said, many of the observations made in this model including sCD163 correlating with CNS and CVD pathology, SIV productive infection of PV macrophage in the CNS and CNS tissues, and neuronal injury of the CNS with SIV infection, as well as the ability to clear CNS infection with ART have been confirmed in humans prior to the advent of ART and in individuals on ART.

This study investigates the distribution of SPION-labeled macrophages within the CNS of normal and SIV-infected animals over time. We found SPION-labeled macrophages primarily in the perivascular space (PVM), meninges, and choroid plexus (CP), identifying that meningeal macrophage had a greater accumulation of SPION than PVM. The data suggest that SIV infection leads to increased accumulation of SPION + macrophages in the CNS, while in non-infected animals, these macrophages traffic out of the CNS. We highlight the significance of SPION-labeled macrophages found in the dcLN, indicating traffic from the CNS, and underscore the need for further investigation into the role of macrophages in viral reservoirs and the impact of ART on CNS inflammation and reseeding of the viral reservoir in the periphery.

## Methods

## Ethics Statement

All animal work was approved by the Tulane National Primate Research Center Care and Animal Use Committee. The TNRPC protocol number is 3767 and the animal welfare assurance number is P0310.

#### Animals and Viral Infection

Sixteen rhesus macaques (Macaca mulatta) born and housed at the Tulane National Primate Research Center in strict adherence to the "Guide for the Care and Use of Laboratory Animals” were used in this study (Fig. 1). Six are non-infected, 10 are SIV-infected with 2 animals used from an additional cohort that developed acquired immunodeficiency syndrome. Animals were intravenously inoculated (i.v) with a bolus of SIVmac251 viral swarm (20 ng of SIV gp41).

#### SPIONs and Dextran

Superparamagnetic iron oxide nanoparticles (SPION) were obtained from Bangs Laboratories Inc (Fishers, IN) (PSCOOHMag/Encapsulated, MEDG001). SPION are iron oxide nanospheres encapsulated in an inert polymer which contains a photoreactive fluorophore. The SPION used have an average particle size of 0.86µm diameter and internal fluorescence (Dragon Green [480/520]). SPION were prepared under sterile conditions using 3 mL of stock solution and 7 mL of sterile, low endotoxin phosphate-buffered saline (PBS). SPIONs were resuspended in 1mL of sterile PBS for a final concentration of 33 mg/mL. Select animals (n = 2) also received fluorescent dextran (RITC-Dextran, 10 KD, Invitrogen, CA) i.c at a concentration of 50 mg/mL.

#### Intracisternal (i.c) Inoculation into the third ventricle

To avoid an increase in intracranial pressure, 1 mL of cerebrospinal fluid was removed from the cisterna magna prior to inoculations of equivalent volume. 1 mL of SPION (33mg/mL) was injected over a 5-minute period.

**Tissue Collection and Processing**: Animals were anesthetized with ketamine-HCl and euthanized with i.v. pentobarbital overdose and exsanguinated. Blood was collected and Heparin Sulfate was administered i.v. and allowed 5 minutes to diffuse. Sodium pentobarbital was administered via intracardiac injection and CSF was collected. Following CSF collection, animals underwent perfusion with 3L of 1XPBS. Postmortem examination was performed by a veterinary pathologist as previously described [33, 34, 52, 85]. Brain (frontal, temporal, occipital, parietal, and choroid plexus) and peripheral tissues (cranial nerves, cribriform plate, nasal septum, brachial plexus, spleen, dcLN, superficial LNs, axillary LNs) were: i) collected in zinc-buffered formalin and embedded in paraffin, ii) fixed with 2% paraformaldehyde for 4–48 hours, sucrose protected and embedded in optimal cutting temperature (OCT) compound for SPION analysis, or iii) snap frozen in OCT without fixation.

**Immunohistochemistry**: IHC was performed as previously described using antibodies targeting CD163 (1:250, Leica (Deer Park, IL)), CD68 (1:100, DAKO (Carpinteria, CA)), CD206 (1:1000, R&D Systems (Minneapolis, MN)), and IBA1 macrophages (1:100, Wako (Osaka, Japan)) [33, 34, 85]. Briefly, formalin-fixed paraffin-embedded sections were de-paraffinized and rehydrated followed by antigen retrieval with a citrate-based Antigen Unmasking Solution (Vector Laboratories, Burlingame, CA) in a microwave (900 W) for 20 min. After cooling for 20 min, sections were washed with Tris-buffered saline (TBS) containing 0.05% Tween-20 for 5 minutes before incubation with peroxidase block (DAKO, Carpinteria, CA) followed by protein block (DAKO, Carpinteria, CA) for 30 minutes and incubation with primary antibody. Following incubation with a peroxidase-conjugated polymer, slides were developed using a diaminobenzidine chromogen (DAKO, Carpinteria, CA) with Harris Hematoxylin (StatLab, McKinney, TX).

**Immunofluorescence**: Antibodies targeting CD163 (NCL-CD163, Leica Biosystems (Deer Park, IL)) and gp41 envelope protein (KK41, NIH (Manassas, VA)) were used on 2% paraformaldehyde (PFA) fixed frozen sections as previously described (Nowlin et al., 2015, Soulas et al., 2011, White et al., 2023). 2% PFA, fixed frozen sections were thawed for 20 minutes at room temperature, unwrapped, submerged in a citrate-based Antigen Unmasking Solution, and microwaved for one minute and forty-five seconds and cooled to room temperature. Slides were permeabilized in a solution of phosphate-buffered saline with 0.01% Triton X-100 and 0.02% fish skin gelatin (PBS-FSG-TX100) followed by a PBS-FSG wash, transferred to a humidified chamber and blocked with 10% normal goat serum (NGS) diluted in PBS-FSG for 40 minutes, followed by a 60-minute primary antibody incubation, washes, and 40-minute secondary antibody incubation. Routine washes were performed and DAPI nuclear stain added for 10 minutes. Slides were mounted using a custom-formulated anti-quenching mounting media containing Mowiol (#475904, Calbiochem; San Diego, CA) and DABCO (#D2522, Sigma: St. Louis, MO) and allowed to dry overnight before being digitally imaged with a Zeiss Axio Scan.Z1. HALO software (HALO v3.4, Indica Labs; Albuquerque, NM) was used for quantification and analysis.

#### Detection and quantification of SPION + macrophages in tissues

SPIONs were detected in the central nervous system (CNS) and peripheral tissues by 1) light microscopy by morphology of the amber SPION beads, 2) Prussian blue iron staining (Sigma Aldrich Iron Stain, St. Louis, MO), and 3) internal fluorescence of Dragon Green. SPION + cells in whole tissue sections were counted manually at 20x by light microscopy (Plan-Apochromat 620/0.7, Olympus; Japan) in a blinded fashion. Whole section tiling, and stitching was done using a Zeiss Axio Imager M1 microscope (Zeiss;Oberkochen, Germany) with AxioVision (Version 4.8, Zeiss; Oberkochen, Germany) using Plan-Apochromat 620/0.8 and 640/0.95 Korr objectives followed by manual annotation of the parenchyma from the meninges into separate regions of interest (ROIs) and tissue area reported as $mm^2$.

#### Viral RNA and Detection

Ultrasensitive SIV-RNAscope with probes for SIVmac251 was used to detect SPION-containing SIV-RNA positive cells within and outside of the CNS as previously described (White et al., 2023). Tissue sections were placed in a target antigen retrieval solution, heated, and treated with protease plus, and a hydrogen peroxide blocker according to the manufacturer’s protocol (Advanced Cell Diagnostics, Newark, CA). SIVmac239 RNAscope probes (Advanced Cell Diagnostics, Newark, CA) were hybridized at 40°C in the HybEZ II Hybridization System. The RNAscope 2.5 HD Assay amplification steps were applied according to the manufacturer’s protocol. Target RNA was visualized through the addition of chromogenic Fast Red A and Fast Red B (Advanced Cell Diagnostics, Newark, CA), and sections were counterstained with hematoxylin (Sigma-Aldrich) and mounted using Vectamount (Vector Laboratories).

### Declarations

**Ethics approval and consent to participate:** This study did not use any human participants or specimens. This study used non-human primates. All animal work was done in strict adherence to the "Guide for the Care and Use of Laboratory Animals" and was approved by Tulane Universities', Tulane National Primate Research Center (TNRPC) Care and Animal Use Committee, New Orleans, LA, USA. The Animal Use Committee approved specific protocols and procedures for animal use based on the Ethical Use of Animals Standards. The TNRPC protocol number is 3767 and the animal welfare assurance number is P0310

**Consent for publication:** Not Applicable.

**Competing interests:** The authors declare that they have no competing interests.

**Data Set Availability:** Data sharing is not applicable to this article as no datasets were generated or analyzed during the current study.

**Funding:** Work in this publication was supported in part by funding from the National Institutes of Health, NINDS, NIH RO1 NS040237 (K.W.) and RO1NS126091(K.W.)

**Authors Contributions:** Conceptualization, K.C.W., and X.A.; Obtained and analyzed data and images, X.A., Z.K.W. J.M., and C.C.M.; writing—original draft preparation, X.A., and Z.K.W.; writing—review and editing, K.C.W., Z.K.W., and P.J.D.

**Acknowledgements:** Not Applicable.

## Figures

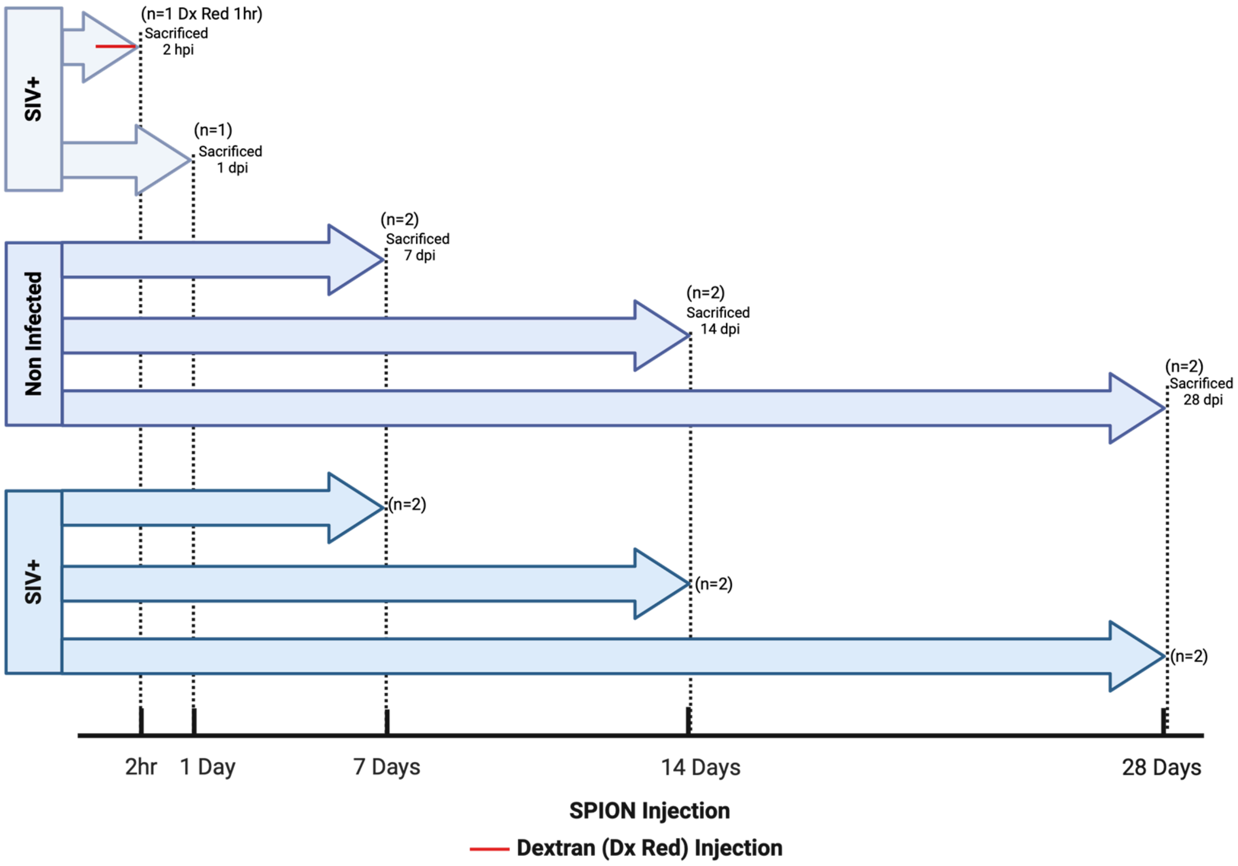

### Figure 1

**Study Design.** Fourteen Indian rhesus macaques were used in this study. Six were non-infected and eight were infected with SIVmac251. An additional two animals that were SIV infected and developed AIDS were used for studies of SIV-RNA in SPION+ macrophages in deep cLN. Macaques received i.c. SPION injection and serial sacrifices with or without dextran dye 1 hour, 1 day, 7-, 14-, or 28- days post-injection. Study design created using BioRender.

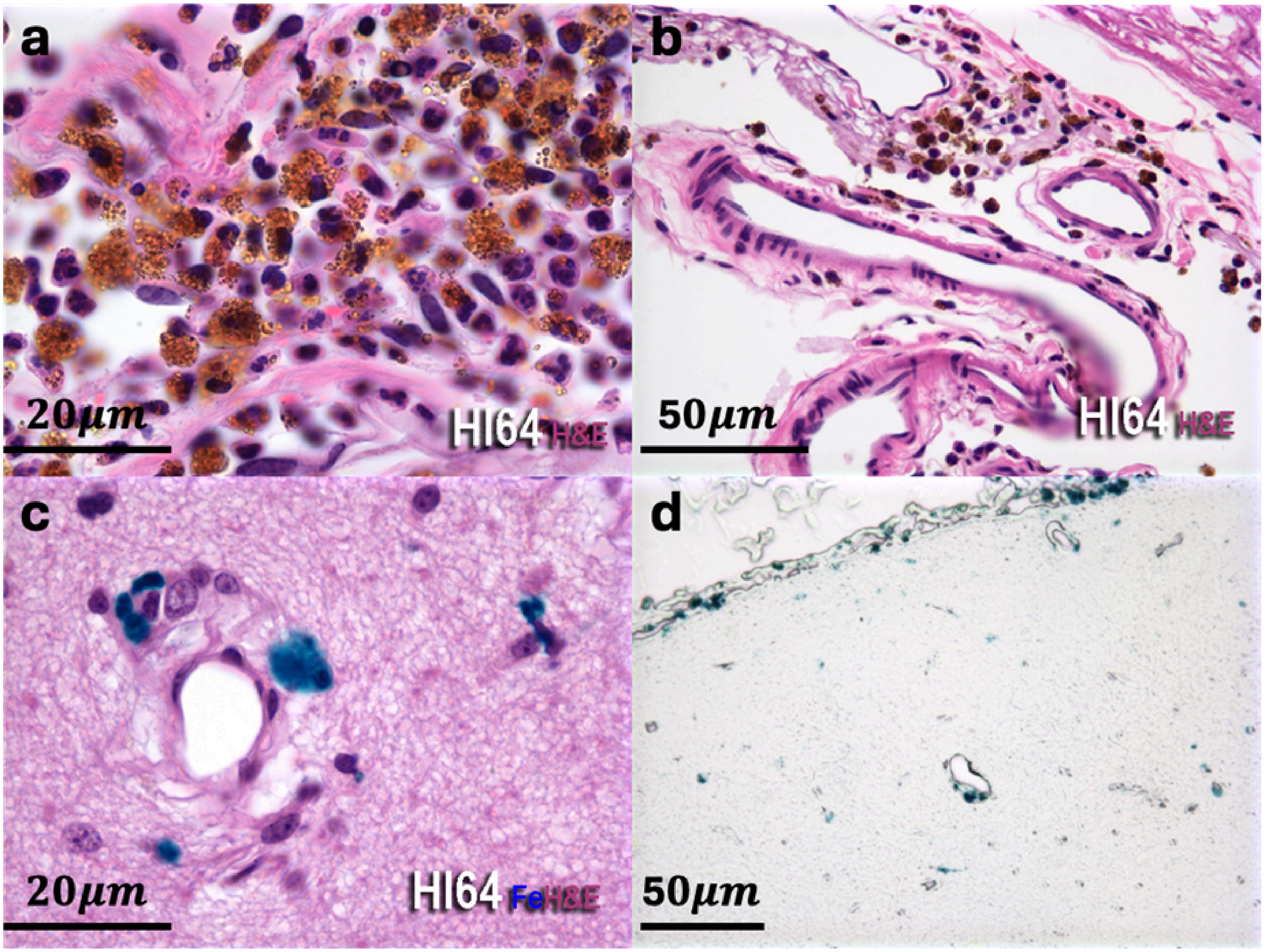

Figure 2

**Distribution of SPION after i.c. injection.** Following i.c. SPION injection, SPION are cell-associated in the CNS (a&b) amber SPION within macrophages in cerebellum meninges, (c) Prussian Blue, iron stained SPION within perivascular macrophages in the perivascular space, and (d) Prussian Blue, iron stained SPION in macrophages within cerebrum meninges and perivascular space.

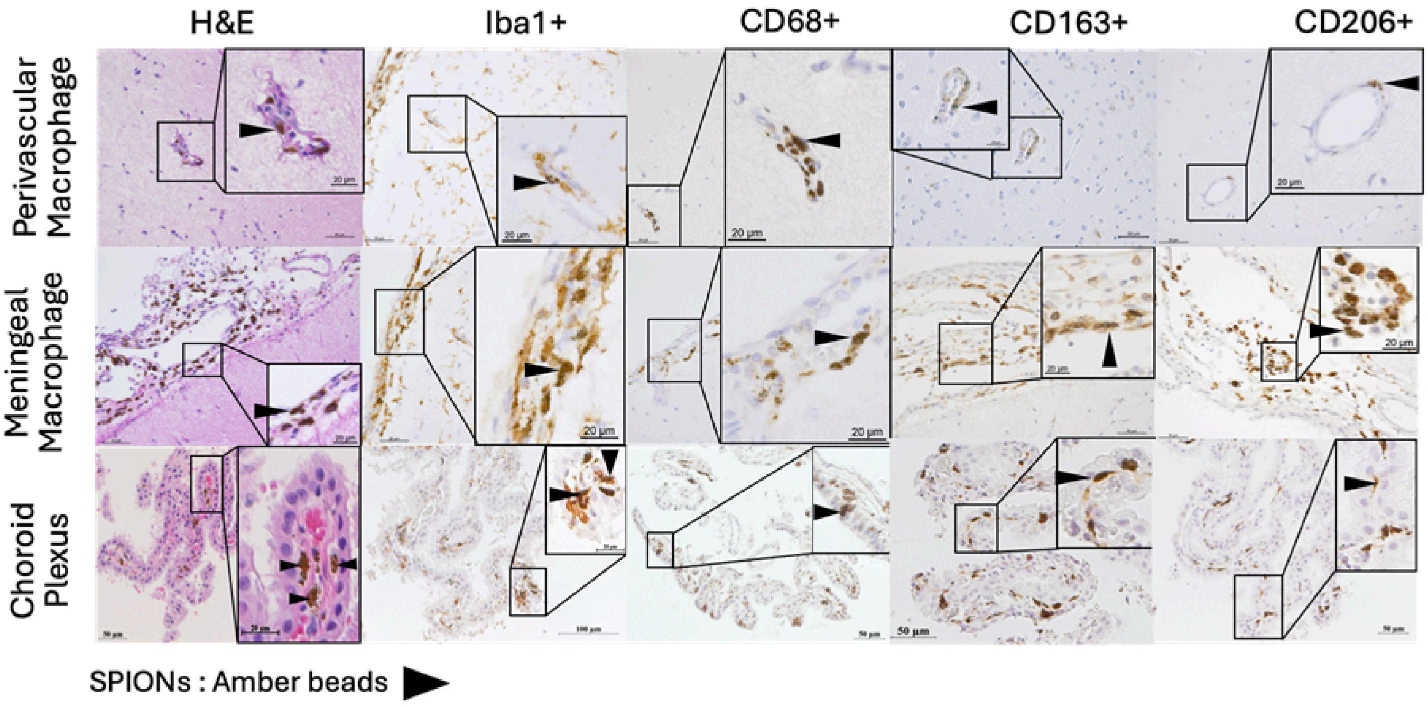

### Figure 3

**SPION localize to CNS macrophage.** SPION (amber beads) within Iba1+, CD68+, CD163+, and CD206+ perivascular, meningeal, and choroid plexus macrophages in the CNS.

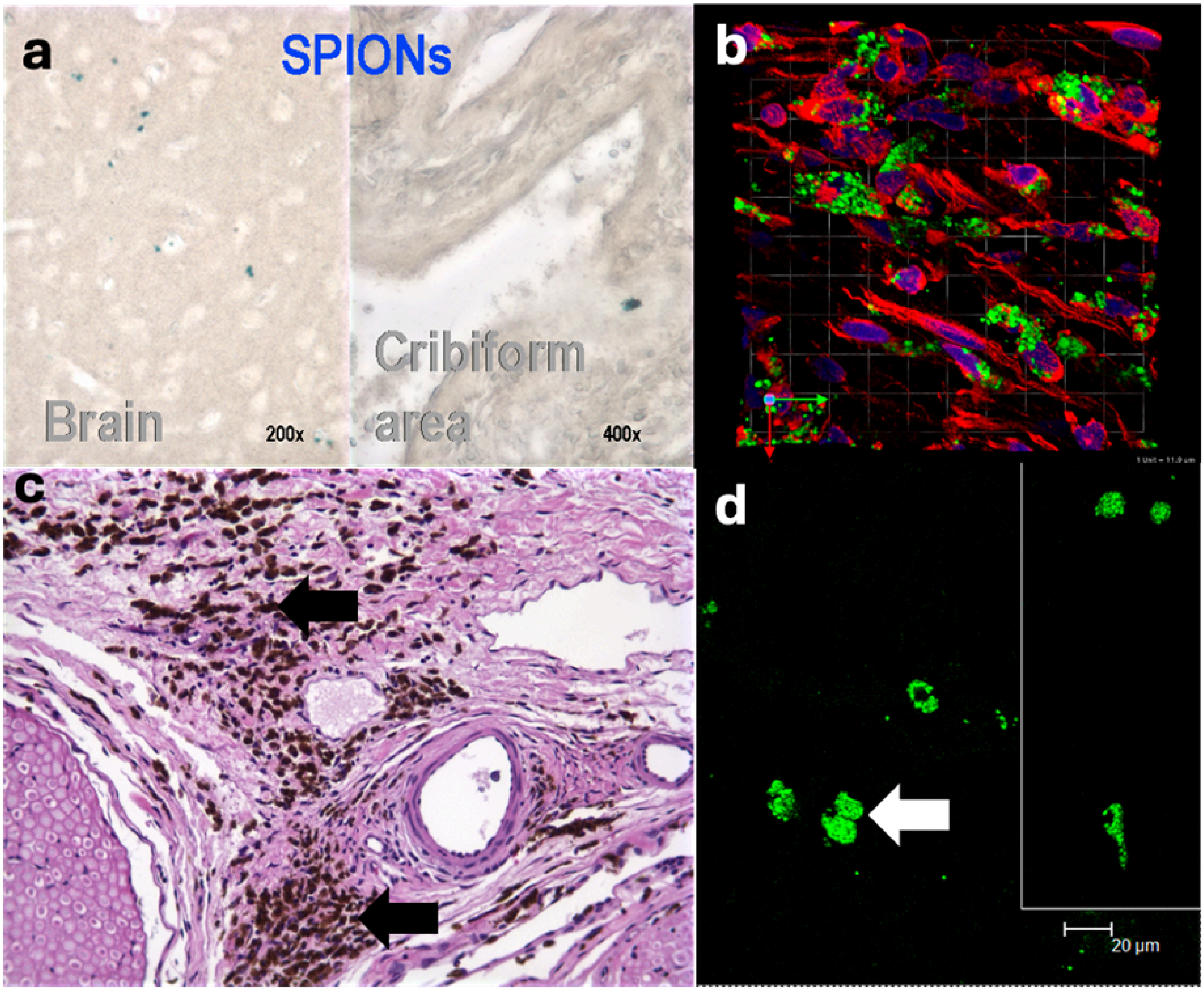

Figure 4

**SPION+ Macrophage outside of the CNS over time.** (a) SPION (Prussian blue) in the brain and cribriform plate area, (b) SPION (green) localized to CD163+ macrophage (Red) within cranial nerves, (c) SPION (amber beads) clustered in the DRGs, and (d) SPION (green) within macrophages in the tonsils.

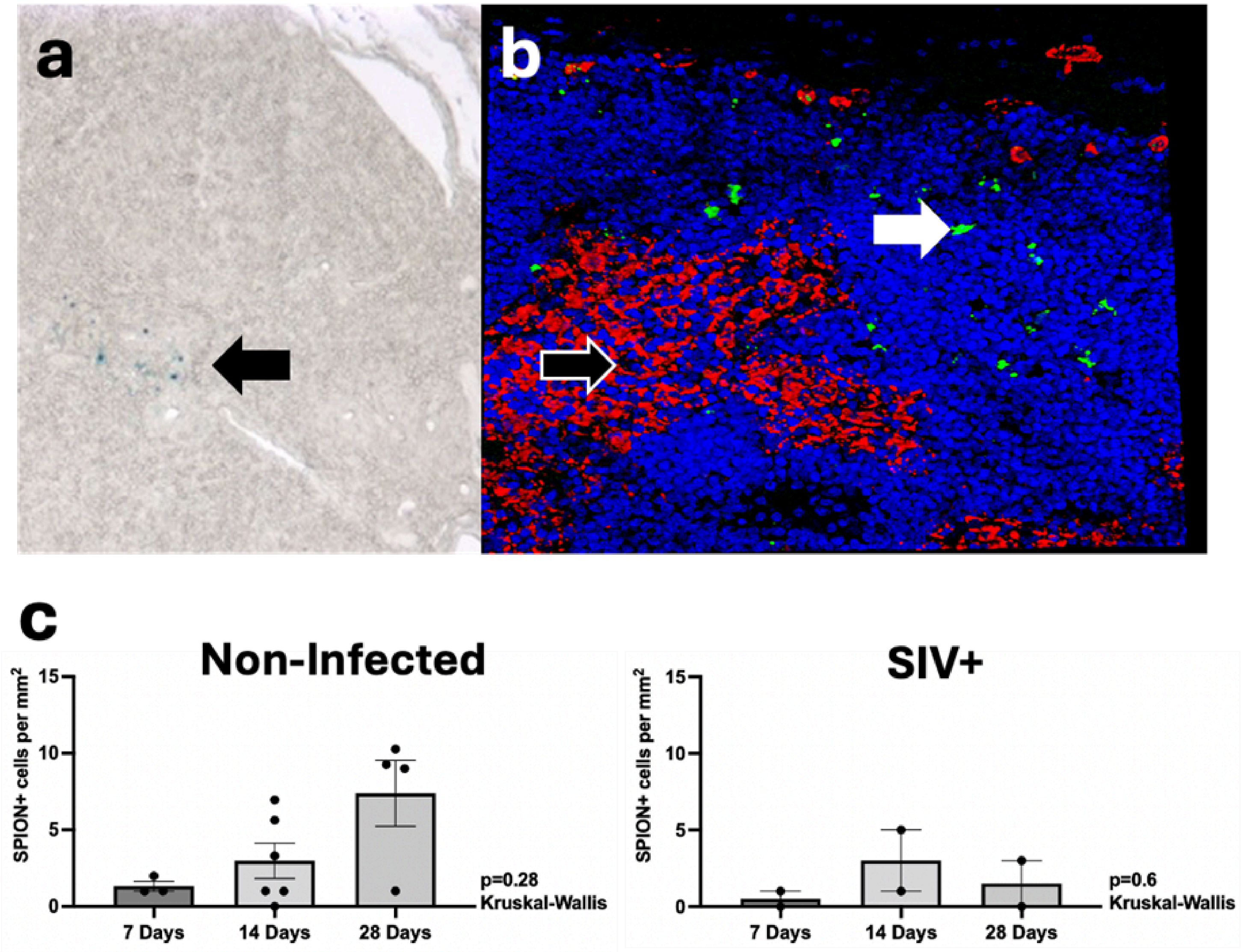

#### Figure 5

**Traffic of SPION+ macrophage out of the CNS to the deep cervical lymph node over time.** (a) SPION (Prussian blue) in the deep CLN 7 days post i.c. SPION injection. (b) Dextran dye (Red, white border arrow) and SPION (green, black arrow) in different regions of the deep CLN post i.c. injection. (c) Number of SPION+ macrophage in the deep CLN post i.c. injection in non-infected and SIV-infected macaques. Each data point represents the cell count from the tissue section examined that is expressed as the number of positive cells per mm$^2$. Prism was used for graphing and statistical analysis using a Kruskal-Wallis nonparametric test.

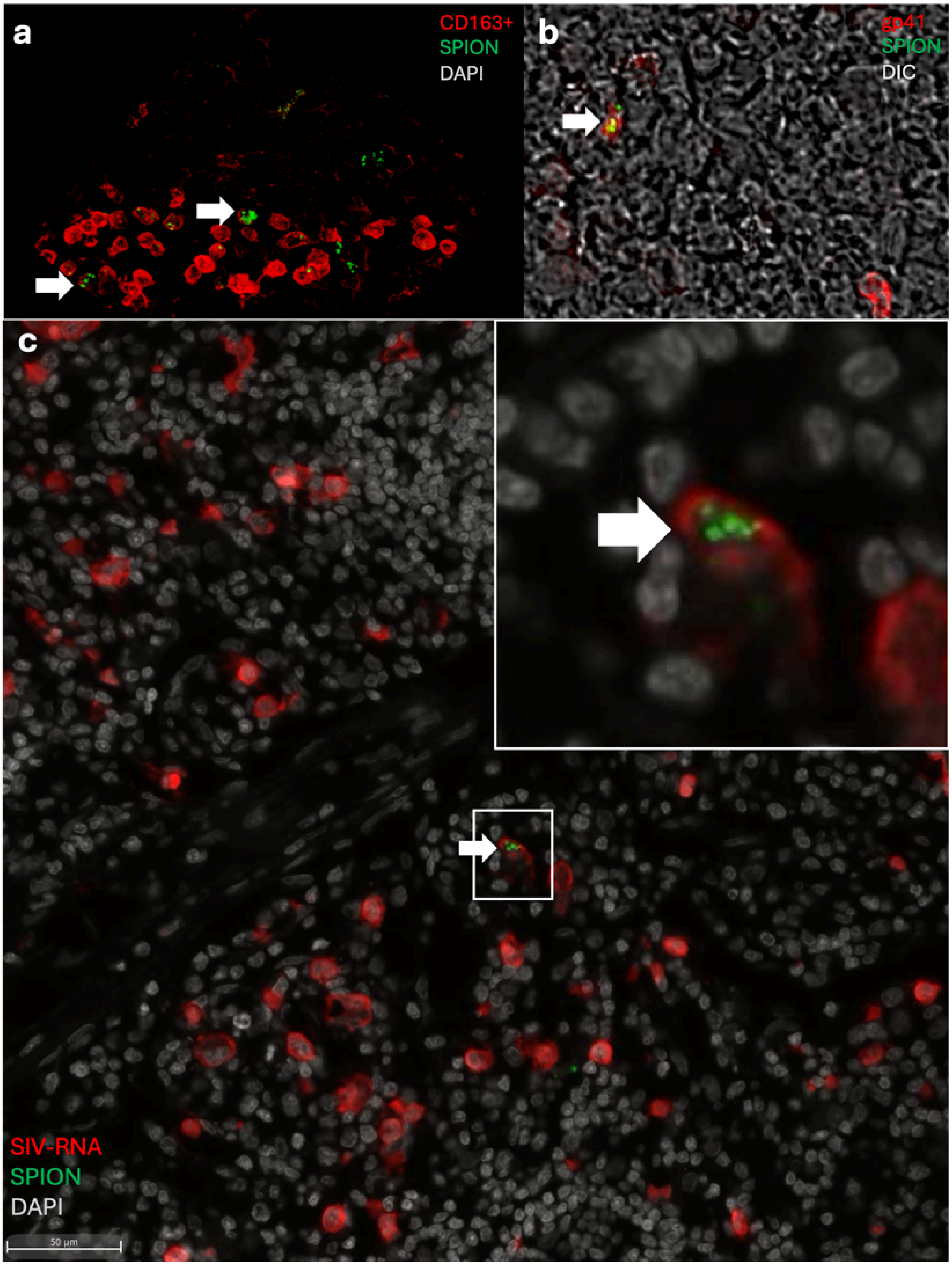

**Figure 6**

**SPION localized to CD163+ SIV-RNA+ gp41+ productively macrophage in the deep cervical lymph node**
(a) SPION (green) localized to CD163+ macrophage (red) in the dCLN following i.c. injection and (b) SPION+ (green) gp41+ (red). (c) SPION+ SIV-RNA+ macrophages in the dCLN in an animal with AIDS and SIVE. Results in this panel are representative of n=2 animals with AIDS.
